# Transactions of the Medical Society of the County of Kings

**Published:** 1865-08

**Authors:** 


BUFFALO

### Medical and Surgical Journal.

VOL. V. AUGUST, 1865. No. 1.

### ART. I.—*Transactions of the Medical Society of the County of Kings.*

### QUARTERLY MEETING, OCTOBER, 1864.

*Cancer.*

DR. HUTCHISON presented a specimen of *malignant disease* of the breast, with the following history: "Mary Ann Daly, aged 38, born in Ireland, sixteen years in this country, married—no children—one miscarriage five years ago. About a year ago the left breast was struck; two or three months afterwards she noticed a hardness in the breast, retraction of the nipple, and induration of the axillary glands of the same side. The induration, when first noticed, was about the same size as at present. There was no discoloration of the integument until about four months ago. About three weeks since, by advice of a druggist, she applied a poultice, which was followed by an ulcer about the size of the thumb-nail, and surrounded by a ring of discolored integument. The glands in the axilla were enlarged to about the size of the end of a man's thumb. The pain in the breast has been sharp, quite severe at times, and again absent for about a week at a time. Thinks she had more pain at night."

The diseased mass was removed in the usual way—no blood vessels had to be tied, a circumstance he had also noticed, where the *tumor* was *enucleated.* The axillary glands were also removed, as they were very much involved. The specimen presents all the *physical* as well as the *clinical characteristics* of cancerous disease. The *flaps* were drawn together by the *continuous silver suture.*

DR. ENOS referred to the difference between *benign* and *malignant* tumors, and enumerated the different diagnostic symptoms. In reference to his own case, he stated that the tumor was not growing very quickly, but was the cause of a great deal of trouble and mental anxiety. He thought it advisable, therefore, to remove it. Did not think it would degenerate into malignant disease.

Dr. Clark remarked that he did not place much dependence upon the *cachectic* appearance of the patients in these cases.

### *Polypus.*

Dr. Colgan presented a specimen of *polypus uteri.* The patient had called some time ago, at his office, and complained of obstinate vomiting, and stated that for the last two or three years she had had frequent hemorrhages from the vagina. Would not submit to an examination, and medication seemed to have no control over the vomiting. Shortly afterwards she gave birth to a seven months' child, and it was during labor that this polypus was discovered, arising from the cervix. In the course of ten or twelve days a ligature was applied, and the mass came away in two days afterwards.

He was sent for again some time afterwards, and found that she could not make water. On examination found a calculus in the urethra.

### *Tuberculosis of the Testicles.*

Dr. Bartlett presented the testicles removed from a patient on the 8th of October, 1864. Four years ago he had gonorrhœa, with more or less inflammation of the testicle. From this, however, as he thought, he had recovered. About two years ago he injured the testicles, and inflammation set in, commencing in the epididymis. From this he never recovered, and after undergoing treatment in several places, he at last found his way into the Kings County Hospital. The testicles were found to be very large and painful, and the scrotum, on the right side, one mass of ulceration. Their removal was determined upon, and the operation was performed by laying the scrotum open, and tying the whole cord. The epididymis, on the right side, was entirely gone, and the seminiferous tubes were filled with tuberculous matter. No spermatozoa were found. In the left testicle, however, some were found, but the greater part of its substance was a mass of tuberculous matter. In the right testicle, the tunica vaginalis was everywhere adherent, and had to be dissected out.

In reply to a question the Doctor stated, that the cord was so short, in consequence of the swelling, that he concluded to tie the whole of it. The contents of the cord were perfectly healthy. Under the microscope the diseased mass of both testicles was found to consist of the tubes of the testicles, increased to double their normal diameter, and filled with the *granular, amorphous matter*, and *shrivelled, granular muclei*, characteristic of tubercle. The Doctor supposed that the patient, when he had gonorrhœa, had epididymitis only on the right side, and that after the disease had subsided, the testicle remained enlarged and sensitive.

---

### MONTHLY MEETING, DECEMBER, 1864.

*Specimens by Dr. S. F. Speir.*

Dr. Speir presented a specimen of *diverticulum from the pericardium.*

The patient was a female, and had been complaining for several years of palpitation of the heart. She died of bronchitis.

On post mortem found the layer of the pericardium absent at the place of diverticulum. There was an arrest of development at this point, and when the fluid accumulated there, expansion took place, and this *diverticulum* was formed. The pericardium was distended with fluid, and the diverticulum extended into the left pleural cavity.

*Atrophy of the Heart.*

In this case, all portions of the body were atrophied ; the heart weighed only four and a half ounces, the natural standard being ten ounces. There was no deposit of fat between the muscular fibres.

*Pyomia.*

The subject of this specimen was a man of intemperate habits. Dr. Minor amputated the leg above the knee; pyomia set in, and the patient died. The principal vein leading from the stomach was occupied by *clots*, some portions of them being surrounded by purulent looking matter. Phlebitis was not apparent. Under the microscope this *matter* was found not to be *pus*, but broken down *fibrinous matter, blood corpuscles*, etc. The *lungs and spleen* were

*tuberculous;* in the *liver* was an abscess, which turned out to be tuberculous.

Dr. Enos supposed that the portion of the *clot*, in the case of pyomia just related, was owing to some want of vitality in the blood.

### *Polypus of the Womb.*

Dr. Hart report a case of polypus of the womb. About six weeks ago he was consulted by a lady, 50 years of age, who was suffering from *uterine hemorrhage*, which was some times considerable, but not at any time profuse. She was married, had never been pregnant, and for the last two years had not menstruated. Her health was perfect; appetite good; slept well, and nervous system in good order. At intervals she was troubled with leucorrhœa, and subject to a serious discharge from the vagina.—Ordered a vaginal injection of tannic acid, and administered iron internally, but the remedies did not produce any benefit. About two weeks ago she noticed a small fibrinous mass come away. A few days afterwards she was seized with expulsive pains, which on the following morning became very severe, and continued for two or three hours, when a substance was thrown off, the pains subsiding immediately thereafter, About five days ago she was again seized with pain, and passed the present substance, which Dr. Speir pronounces polypus.

Dr. Speir stated that the tumor was composed of cells and corPuscles, and was undergoing fatty degeneration.

---

### REGULAR MEETINGS, MARCH AND JUNE, 1865.

### *Action of Medicines on the Blood-Vessels.* By R. Cresson Stiles, M. D.

The following experimental researches were undertaken with the desire of extending, as far as possible, the influence of the anatominal generalizations of Bichat and the discoveries of modern histologists into the domain of practical medicine. The field of research was chosen as near as possible to that of practical medicine, in order that the results of experiment might be controlled by the experience of numerous observers among practitioners, instead of affording interest to the professed cultivators of abstract physiology merely, whose numbers are few, and with the tendency of whose pursuits whatever there is of magisterial influence and of practical conservatism in medical authority has but limited sympathy. Physiology is not far in advance of the position which chemistry held before the time of Lavoisier, but the movement which is drawing within the domain of the *Science of Vital Dynamics* the dependent science of Pathology, (dependent, if legitimate scientific dependence exist at all,) which leads the physician to seek in diseases the affections of an organism re-acting in accordance with definite laws, rather than entities whose slightest variations in form and shade, in flying clouds of symptoms, are to be depicted in endless and useless detail, must affect also the classification and employment of therapeutic agents.

The several tissues of the body manifest varied and delicate differences of re-action to the agents employed in their study. These differences, due to molecular constitution, they doubtless possessed, with others of still greater delicacy, while forming a part of the living organism, rendering them subject to the variations of nutrition or function, which are the essential elements in all medicinal action. The influence of sulpho-cyanide of potassium and of upas upon the voluntary muscles, that of strychnine upon the afferent nerves, and that of wourara upon the motor nerves, give proof of relations subsisting between these substances and tissues respectively which other tissues do not share, or in which they participate to but a slight extent. The power of carbonic oxide gas to paralyze the blood corpuscles, rendering them inert in hæmatosis, and that of a temperature of 115° Fahrenheit to paralyze and make rigid the voluntary muscles, while the same temperature leaves the motor nerves intact, lend further proof of the independent vital re-actions of the different anatomical elements of the body. That these relations should be more familiar to physiologists than to practitioners is simply because they have been studied by the former; that many of the most familiar and useful articles of the materia medica have like relations I propose to show. Similar considerations are applicable to the generation of diseases. The development of the Trichina Spiralis in the striated muscles exclusively, its presence ceasing, as I have seen it, with the upper third of the œsophagus, leaving the remainder of an apparently homogeneous canal uninfested, is a type of the mode in which other less vitalized morbific agents seek out certain tissues of particular physical or molecular constitution upon which to exert their activity.

Some of the most valuable revelations of experimental physiology have resulted from the study of the nervous system. The demonstration of the separate motor and sensory endowments of the anterior and posterior spinal nerves by Bell and Magendie, the determination of the powers of the principal centres of the cerebro-spinal system by Marshall Hall, Flourens and Longet, the recognition of the influence of the sympathetic upon nutrition, calorification, secretion, and the activity of the nervous centers by Bernard and Brown-Séquard, are examples of what has been accomplished in this field of research. It is characteristic of the nervous system, that its functional activity is brought into play by the simplest physical influences, rendering it particularly suited to experimental investigation. To the student of experimental physiology, or to the pathological anatomist, the fact cannot fail to present itself, that the office of the nervous system is rather of a passive nature, serving to call into action and regulate processes of far greater independence; both are led to regard the nervous system as an instrument whose wonderful harmonies are evoked by the play of forces external to itself. Not only has experiment shown that in death from inanition, while the blood loses over seven-tenths of its weight, and the muscles over four-tenths, the loss of the nervous system is less than one-fiftieth; but when we witness on post-mortem examinations the almost uniform complete integrity of the nervous system amid the wreck of other systems and the waste and decay of the rest of the body, and after death accompanied with the most violent nervous commotions, we are forced to regard the nervous system as an instrument of sluggish nutritive changes, rather acted upon by the blood and the organs which it supplies, than itself generating its own forces and possessing the active nutrition necessary to such manifestations of energy. The nervous system has too long given sanctuary to all manner of fugitives from physiological law.

Except from our own individual sensations, we know nothing of nervous action but by muscular action, and it becomes exceedingly important therefore to determine whether the nervous system is not merely one of the instruments through which muscular activity is aroused, or whether physical and chemical agents do not exercise a direct and immediate influence upon muscular tissue through the medium of the blood.

Both Harvey and Haller denied the irritability or muscular contractility of the arteries. The contractile phenomena presented by the blood-vessels were believed by Bichat to be due to *tonicity*, an endowment different altogether from muscular contractility. "Respecting the final arterial ramifications," says Magendie, in 1822, "as the vessels which constitute them are so small as to be invisible, at least in a state of health, no one can either affirm or deny that they are irritable. It would follow from analogy, however, that they have no sensible movement. In cold-blooded animals, it is easy to see the blood circulating in these vessels, and even passing into the veins, but these same vessels give no sign of contraction." These views, thus attested, may be considered as those of the profession generally, till completely reversed by the revelations of the microscope. Since the discovery of the elements of the non-striated muscular tissue or fiber-cells by Kölliker, in 1847, and their recognition in the arteries and arterioles, the question has been set completely at rest. Numerous experimenters have studied the influence of mechanical irritation, of galvanism, of heat and cold, and of various medicinal substances upon the caliber of the vessels, and the rapidity of the circulation in the transparent tissues of the lower animals. The most real and decided of the recent triumphs of experimental physiology have been the result of researches upon the influence of the nervous system upon the circulation, as regulated by the muscular tissue of the blood-vessels, placing under the control of the experimenter the functional activity of various organs, and enabling him to produce at will the fundamental pathological phenomena of inflammation, hypertrophy, atrophy, and degeneration. But it is impossible to resolve these results into their elements. The solution of the simplest physiological problem is rendered difficult by the complexity of the conditions under which it is presented. Our present problem is to determine the influence of certain agents upon the living muscular tissue of the blood-vessels, so as to be able to assign to this influence the part it plays in the involved phenomena of the action of these agents when used as remedies, or otherwise acting upon the human system; or, given a medicinal article, to determine whether, when brought into contact with the muscular tissue of a living artery, it produces its contraction, relaxation, paralysis, or a succession of these states; in what proportions used, and under what conditions these effects may be looked for with certainty. A step only towards its solution is still a step in advance.

The earliest and simplest observations made in this direction were on the obvious effects of cold and hæmostatic applications. By such observations, and by experiment, Hunter distinguished the elasticity of the arteries from their contractility, and recognized the persistence of the endowment of irritability long after death. He divided longitudinally arteries of different caliber, and determined, by measurement, the part in their contraction due to elasticity, and that due to physiological contractility, recognizing, before the microscope had shown that the smallest arteries are entirely wanting in elastic tissue, while their muscular tunic is well developed, that contractility has the largest influence upon the smallest arteries. He showed that contractility had no influence on the length of the arteries before minute anatomy had demonstrated the circular arrangement of their contractile elements. Kölliker experimented on the arteries of an amputated limb with a galvanic current, and produced contractions and constrictions of the popliteal and posterior tibial for an hour after the operation.

I have already alluded to experiments on the circulation in the transparent tissues of the lower animals, as seen by the microscope. These are now of vulgar performance, but it was not so when Schwann announced that he had produced successive contractions to one-third of their diameter in the mesenteric arteries of the toad by successive applications of cold water; or Ed. Weber reported having reduced the mesenteric arteries of the frog to one-sixth of their original diameter by an interrupted galvanic current. Numerous medicinal substances have since been tested in this manner, but the direct effect of the agents employed upon the muscular tissue is here involved with that upon the nerves, either directly or through reflex action, with the results of endosmose and exosmose, and with numerous other complications; and both the classes of experiments I have mentioned lack the quantitative determination, which is the surest basis of scientific generalization. Resort must be had to simpler methods.

The quantities of a liquid discharged through inert tubes of a caliber, such that the effects of friction are inconsiderable, are, other conditions remaining the same, as the squares of the diameters of the tubes As the tubes diminish in size, the effects of friction become more important, till, in capillary tubes, according to Poiseuille, the quantities discharged are inversely as the length of the tubes, and directly as the fourth power of their diameters. It follows, therefore, that a cause which would reduce the diameter of a capillary tube one-half, would reduce the amount of liquid discharged to one-sixteenth. Obviously the most accurate method of estimating the effect of delicate influences npon the caliber of the blood-vessels is that of measuring the amount of liquid they permit to pass through them under given conditions.

Poiseuille observed, also, that minute differences in the constitution of the liquids exerted a decided influence upon the rapidity of their flow through capillary tubes. Thus, the flow of serum was retarded by alcohol in such quantities as might be supposed often mingled with the blood during life, and that of the alcoholic serum accelerated by ammonia, and he argues that the beneficial effects of ammonia in drunkenness are due to this antagonistic influence. On injecting acetate of ammonia into the blood of a horse, he reduced the time of the round of the circulation from twenty-five and thirty seconds to eighteen and twenty-four seconds, and increased it to thirty-five and forty seconds with chloride of sodium. Iodide of potassium, nitrate of potassa, and chloride of ammonium, increased the rapidity of the circulation in animals, and of the liquid flow in inert tubes. Although his deductions from these experiments are manifestly unwarrantable, the influences which they reveal are important, and should not be neglected; they should not be allowed to vitiate the result of experiments on muscular irritability.

After numerous trials of apparatus and liquids in the endeavor to avoid sources of error, the following arrangement was adopted for a course of systematic experimentation. A reservoir is placed four feet above the operating table, to the bottom of which is adapted a long tube of caoutchouc terminated by the nozzle of a syringe for anatomical injection. The tube is coiled in another reservoir on the table, of a capacity of several gallons, into which water at any required temperature may be introduced, and lose heat slowly. The temperature of the liquid in this reservoir is that of the liquid discharged from the mouth of the tube, when the upper reservoir has been filled and the current allowed to flow. It is not raised above 110° Fahrenheit, nor allowed to fall below 100°. The liquid most used was a solution of sugar, of a specific gravity of from 1025 to 1030, perfectly neutral, not abstracting water from muscular fibers, nor imbibing them, but permitting them to maintain their irritability for a long period when immersed in it; not affecting the artery experimented on in its passage through it otherwise than mechanically. Blood and serum from the lower animals were employed occasionally to control the results obtained with the saccharine solution. The arteries experimented on were chiefly those of the umbilical cord, which the lying-in ward of a large hospital furnishes in abundance, and which are peculiarly adapted for the purpose. Other arteries were employed, and even whole organs of the lower animals, recently killed, were connected by their arteries with the apparatus, for the purpose of studying the capillary circulation.

The lower reservoir serves also as a water-bath, in which vessels containing the medicated solutions are immersed, their temperature being also that of the liquid flowing through the artery. The artery having been adapted to the tube, and the rate of flow through it measured, the current is arrested, the artery is suspended for a given time in the medicated solution, and the rate of flow is again measured. Or, the rate of flow having been measured, the substance to be tested is mingled in given proportion with the liquid in the upper reservoir, and thus allowed to act on the interior of the artery as in the circulation of the blood.

The arteries of the umbilical cord contain no elastic tissue, blood vessels, nor nerves, but their muscular tunic is remarkably developed, imbedded merely in a loose connective tissue. No other large blood vessel presents so completely the simple, uncomplicated phenomena of muscular contractility. Virchow, in his Cellular Pathology, has given a true description of the umbilical cord, showing the organized structure of the so-called gelatine of Wharton, and its likeness to the vitreous humor of the eye. He mentions the extraordinary development of the muscular coat of the umbilical arteries, and denies the existence of other blood vessels than the main channels in its structure. Prof. Simpson thus resumes the result of his investigations on the structure of the cord and placenta:—"Into the composition of these parts no capillaries, vasa vasorum, lymphatics, nor nerves are found to enter; hence, in human anatomy, we have these organs forming a large mass, and weighing on an average about two pounds, presenting a type of structure resembling that of some of the inferior zoöphytes." I have sought very carefully with the best powers of the microscope for nerves in the umbilical arteries, and have witnessed no appearance which would give the least suspicion of their existence, but the muscular fibers have their typical character and micro-chemical re-actions. They may be beautifully isolated by maceration in dilute nitric acid, and, not being associated with elastic tissue, present the appearance they offer in the minutest arteries of the adult.

The arteries of the cord contract immediately on separation from its attachments, so that it is with difficulty that a liquid can be forced through them. In this state they appear like impermeable cords of a line in diameter. Hunter observed that they would retain this appearance for several days, till incipient decomposition produced complete relaxation. When relaxed completely, they are like flat bands of two lines in breadth, and transmit liquids in copious streams. The persistence of their irritability, though most probably not greater than that of adult arteries, fits them for prolonged experimentation.

### 1st.—*On the Influence of Different Degrees of Heat on the Caliber of the Blood Vessels.*

The current flowing through a piece of umbilical artery was gradually heated, and as the following degrees, measured by a delicate Centigrade thermometer held in the stream, were reached, the following quantities were discharged in periods of three minutes each:

| | | | |
|---|---|---|---|
| At 20 Centigrade, | 68 | Fahr. | 3 fluid drachms. |
| At | 86 | " | 4 " " |
| At | 91 | " | 5½ " " |
| At 34° " | 93.2 | " | 7½ " " |
| At " | 98.6 | " | 11 " " |
| At 46 " | 114.8 | " | 5 fluid ounces. |

I have not determined the law of dilatation under different degrees of heat and varying rapidity of transition, but this series of observations proves, as did numerous others, that arterial dilatation is increased with elevation of temperature, and that the vascular irritability is most marked in the vicinity of blood-heat. An exceedingly interesting point is the limit of dilatation and the complete relaxation and paralysis of the muscular tissue at 115° Fahrenheit. This I have established by most careful observations, and the proof that the same phenomenon occurs in living animals also, I have given in an article on sun-stroke in the *Boston Medical and Surgical Journal* for June, 1864. When the temperature of the blood in rabbits has been raised to this point, the blood vessels of the ears, turgid with dilated vessels and rapidly circulating blood, refuse to contract under the influence of cold and galvanic currents; when this symptom manifests itself, death soon ensues.

Water was heated to 55° Centigrade, and, as it cooled, portions of umbilical artery at the temperature of the room were immersed in it, at intervals of two degrees of temperature and for a minute of time. At 55° and 53° Cent., the artery became perfectly flaccid; at 51°, the flaccidity was not so complete, the artery possessed the slightest degree of tonicity; at 49°, the artery coiled up on immersion, and remained in a state of rigidity after it was withdrawn; at 47° it coiled up on immersion, but on exposure to the cold contracted more firmly; at 45° and 43°, no marked contraction ensued during immersion, but followed its withdrawal and exposure to the cold. These experiments prove that what is true of the striated muscular fiber, holds good also with the non-striated fiber of the arteries, both becoming rigidly contracted and paralyzed by a sudden elevation of temperature to the vicinity of 115° Fahr.

In these experiments, no *active dilatation* could be perceived.

The artery simply yielded to pressure from within, or to mechanical distention.

Evidence that heat has the power of relaxing the muscular coat of the finest arterioles was gained by experimenting on whole organs with the defibrinated blood of the lower animals. When a column of blood, four feet in height, connected with the artery of an organ—a kidney, for example—could not force a single drop from the veins, it was sufficient to immerse the organ in water at or above 100° Fahr., to admit free circulation through its capillaries, the rapidity of which rose with the elevation of temperature.

2d.—*On the Effects of Medicinal Substances on the Caliber of the Blood Vessels.*

The medicinal substances subjected to experiment were chosen from the class of stimulants and sedatives, as most likely to manifest a direct influence on the muscular coat of the blood vessels. In Wood's classification of the Materia Medica, the systematic stimulants proper are divided into the arterial, nervous, cerebral and spinal, and the systemic sedatives into the arterial, nervous and cerebral. Representatives of each of these classes were tested, and proof secured that many of them are directly upon the blood vessels.

1. The *Arterial Stimulants*, according to Wood, are capsicum, oil of turpentine, certain ammoniacal preparations, of which carbonate of ammonia is chief, and phosphorus. Of these, carbonate of ammonia was deemed most worthy of study, from its undoubted medicinal powers, from the interest it has excited in theories of uræmic convulsions, of the coagulation of the blood, and of the causation of a low grade of fevers.

In one series of experiments with this substance to the liquid flowing steadily from the artery, at the rate of four and one-third fluid ounces every two minutes, the carbonate was added in the proportion of fifteen grains to the pint. The flow soon reached five fluid ounces in the same period, and as the ammonia, or amono-carbonate, evaporated from exposure to the air in a thin stream at the temperature of 100° Fahr. (the liquid having been poured back into the upper reservoir after each observation,) the quantity discharged fell to three and one-third fluid ounces. On adding an additional fifteen grains, the quantity rose to four fluid ounces. Here the play of the artery, under the varying doses of the carbonate, proved that we were dealing with physiological irritability, and not with mere physical or chemical phenomena.

To control these results and eliminate the influence shown by Poiseuille to be exerted by ammonia to hasten the current of liquid in inert tubes, arteries were immersed in solutions of the carbonate of different strength, the liquid flowing through them being the simple saccharine solution. An artery which discharged one and five-sixths fluid ounces per minute before it was suspended in a solution of the carbonate of the strength of a drachm to the ounce, after five minutes' immersion, discharged sixteen ounces per minute. An artery which, before immersion, discharged two ounces per minute, after immersion for three minutes in a solution of the strength of ten grains to the ounce, discharged eight ounces per minute. An artery which, before immersion, discharged two and one-sixth ounces per minute, after immersion for twenty minutes in a solution of the strength of two and a half grains to the ounce, discharged five ounces per minute. From the first experiment there resulted complete paralysis of the artery; from the others, relaxation merely, with conservation of irritability. Although the proportions of the carbonate were greater than those of the medicinal doses to the amount of blood in the circulation, still the arteries are undoubtedly more sensitive in a living animal than they were in these experiments.

The fact that ammonia possesses the power of relaxing the arteries having been proved, it remained to trace its influence upon the volume of the arteries, and the force and frequency of the pulse when administered as a remedy in disease. This study led me to several unexpected results. I found that I could administer thirty grains every fifteen minutes for an hour without the slightest influence on the pulse; that I could in no case produce its excitement, and that in several cases the result was its actual depression. It was strange that the medical authorities who testified to the stimulating properties of the sesqui-carbonate, when given in doses of ten grains every two hours, should have omitted to mention that it might be administered in doses of thirty grains every fifteen minutes, with no appreciable effect on the system.

It was a disappointment to find the conclusions I had drawn from my experiments in the laboratory, falsified by further experiments in the wards. I had not taken into consideration that carbonate of ammonia in solutiou is decomposed, according to Wood, "by most acids," "and by most salts with excess of acid," "by potassa, soda, and their carbonates; by lime-water and magnesia," and "by the soluble salts of lime;" that the gastric juice contains free hydrochloric and lactic acids, and that this secretion is seen to be poured out abundantly when the stomach of one of the lower animals is touched through a fistulous opening by a rod dipped in an alkaline solution; that, should the carbonate escape decomposition by the acids of the gastric juice, the alkalies and their carbonates would meet it in the blood, and destroy it often, as rapidly as it should be absorbed; that, should the carbonate be absorbed more rapidly than it could be decomposed by the gastric juice and the blood, it would probably not enter the arterial blood at all, but that portion which continued to be volatile, would escape from the system by the lungs, as sulphuretted hydrogen is known to do when sulphur-waters have been taken into the stomach. The systemic influence of the carbonate would therefore be that of a more stable compound of ammonia, as the muriate for example; its local inflnence would be one of irritation to the stomach, if not sufficiently diluted, and perhaps one of the free ammonia in the lungs. After taking a drachm of the carbonate, I caused all the air I expired for fifteen minutes to pass, in fine bubbles, through a linen tissue, kept saturated with dilute hydrochloric acid. The evaporated liquid yielded a notable amount of sal-ammoniac. As the linen had been previously soaked and washed in dilute hydrochloric acid, the ammonia could have had its source only in the expired air, and so large a proportion could not have been an ordinary physiological phenomenon. I concluded therefore that when large doses of carbonate of ammonia are taken, free ammonia escapes by the lungs. It is possible that the decided benefit with which the carbonate is administered in certain pectoral diseases, is due to this cause. In the experiments of Frerichs on uræmia, the injection of carbonate of ammonia into the blood was followed by convulsions, and the abundaut escape of ammonia from the lungs. The question of the amount of ammonia expired, after large doses of the carbouate, should be settled by competent chemical authority.

The speedy precipitation of large and abundant crystals of the ammonio-magnesian or triple phosphate from the urina sanguinis, which, before the administration of the carbonate, had no such tendency, proved that an ammoniacal compound had found its way into arterial blood; but my experiments had shown that the stable ammoniacal compounds—the chloride, at least—had no influence in dilating the arteries, and thus the results of clinical observation and those of experiment were reconciled; moreover, if I inspired for five minutes the air passing into a flask containing a solution of carbonate of ammonia, the excitement of the pulse was immediate and very manifest—but so was the irritation of the lungs.

That a general relaxation of the muscular tissue of the blood vessels must produce those febrile phenomena called by physicians "general stimulation," can be shown to follow from well-known physiological laws.

The inconsistencies of the nervous theory of fever have been acknowledged by some of our best pathologists, who still cling to the doctrine of Cullen. How they can explain the effects of counter-irritation by the admission that there is in the body only a "certain amount of nervous influence," which when called to one part must forsake others, and yet trace to nervous influence the phenomena of fever, which they define as "a general disease affecting all the functions," has always seemed to me incomprehensible. The truth is, that a general excitement of the system cannot be a nervous manifestation simply. If one sense is developed or exercised, it is at the expense of the growth or activity of the other senses; the activity of the cerebrum calls off energy from the automatic nervous apparatus; pure nervous diseases are of a local character; all nervousness is non-febrile; severe injuries affecting the nerves are not immediately followed by fever. These and other facts of the same bearing are too patent, one would think, to the daily observation of physicians, to permit them to lay much stress on the accepted nervous theory of fever. In all nosologies, the pyrexiæ and neurotica form as distinct classes as nosological classification allows.

[To be Continued.]

